# Effect of Family Empowerment on Asthma Control in School-Age Children

**Published:** 2018-01

**Authors:** Zahra Kashaninia, Zahra Payrovee, Reza Soltani, Seyed Alireza Mahdaviani

**Affiliations:** 1 Department of Pediatric Nursing, Faculty of Nursing and Midwifery, Iran University of Medical Sciences, Tehran, Iran,; 2 Pediatric Respiratory Diseases Research Center, National Research Institute of Tuberculosis and Lung Diseases (NRITLD), Shahid Beheshti University of Medical Sciences, Tehran, Iran,; 3 Department of Statistics, University of Social Welfare and Rehabilitation Sciences, Tehran, Iran

**Keywords:** School-age children, asthma, family empowerment, control

## Abstract

**Background:**

Recent surveys have showed that asthma control still remains suboptimal. Family members have an extensive impact on the level of asthma control in school-age children. Family empowerment has a positive impact on the quality of life of school-age children with asthma. This study aimed to determine the efficacy of family empowerment on asthma control in school-age children.

**Materials and Methods:**

Forty-five children with asthma (6–12 years) and their parents were enrolled in a pediatric asthma clinic during their follow-up visits. The family empowerment program consisted of self-directed educational material, lectures (a nurse-focused format), group interaction (a learner-focused format), group discussions, and demonstration of educational films. The primary outcome was change in asthma control measured by the C-ACT questionnaire.

**Results:**

In this study, 45 patients were enrolled and randomly divided into two groups: intervention (n=23) and control (n=22). Demographic variables including age and sex were not significantly different between the two groups. There were no significant differences in pre-test asthma control scores between the intervention and control groups at pre-test (p=0.82). However, there was a significant difference in asthma control scores between the intervention and control groups at post-test (p<0.001). In the intervention group, in which children experienced family empowerment, asthma control scores were significantly higher at post-test compared to pre-test (p<0.001).

**Conclusion:**

Family empowerment significantly improved asthma control in school-age pediatric patients. This program could be proposed for proper asthma control and complication-reducing management of the disease. This program is recommended more broadly for other age groups.

## INTRODUCTION

Asthma is a chronic decrease that impacts the quality of life of 6.5 million children and their families annually. The recurrence of asthma symptoms and asthma attacks are related to the degree of asthma control. The variability of asthma trigger factors makes asthma attacks and symptoms unpredictable and stressful for the family. Despite the continued development of improved treatments for asthma, recent surveys have showed that asthma control still remains suboptimal (1). The chronic nature of asthma and long-term experiences with children with asthma make parents vulnerable as the result of a stressful life that impacts their daily life activities and job. In fact, the asthma severity of children aged 7–17 years negatively affected caregiver’s and patient’s quality of life and socio-demographic characteristics (2). On the other hand, caregivers also have an extensive impact on the level of asthma control (3). Efforts to improve the quality of care for asthma in pediatrics have resulted in growing evidence supporting the fact that the inclusion of family empowerment is necessary in asthma control protocols (4).

Elementary school age (6–12 years) is an important time in the life of children with asthma as well as their families. At this age, children commence school and spend increasing amounts of time away from home, leaving them exposed to more triggers and stresses. In addition, at school age, the management of asthma is performed by both the family and patient instead of the family alone. Therefore, family and patient interaction becomes more relevant in the progression of asthma control. In order to manage asthma during this period, parents need to understand various asthma treatments, and learn to monitor symptoms and respond quickly to changes in condition. Therefore, efforts should be made to develop parents knowledge of asthma management in their family life and also improve children self-management responsibility (5).

There is no consensus on the best model for asthma education for families. Current guidelines recommend family empowerment through education at every opportunity across the health care continuum (6). The National Asthma Education and Prevention Program, led by inpatient asthma nurse practitioners (IANPs), combines several teaching strategies for parents (7).

Family empowerment refers to helping the family of patients attain mastery in self-modification. The family’s role is the same as a group helping its members to develop in different aspects of their life. Family empowerment improves all family members’ skills and knowledge in controlling asthma; part of the family empowerment course involves training family members on how to use inhalers, respiratory assist devices, and activity and nutrition of children with asthma. Participation in family empowerment programs has a significant association with multiple outcomes. Family empowerment has showed a positive impact on the quality of life of school-age children with asthma (8).

Nurses have a major role in family empowerment, which is attributed to the nurse-patient-family relationship that enhances health and prevention teaching. Management of asthma by an asthma nurse has been proven to be effective for asthma control because of proper education that the nurse provides the family or patient (9). Nurses can use strategies that assist the family through education for decision-making, setting family-selected behavioral goals, and providing information about the importance of their role in disease management (10).

The prevalence of asthma symptoms in Iran is higher than that estimated by international reports. This implies that asthma prevention and control should be planned through family and patient education (11). The useful components of the education programs remain to be elucidated (12), and the effectiveness of family empowerment on asthma control in school-age children has not been studied in Iran previously.

This study aimed to determine the efficacy of family empowerment on asthma control in school-age children with asthma.

## MATERIALS AND METHODS

### Study Design

The study used a quasi-experimental design, and forty-five children with asthma (6–12 years) and their parents were enrolled in the pediatric asthma clinic in Masih Daneshvari Hospital during their follow-up visit. Participants were recruited using convenience sampling and were randomly divided into two groups: intervention (n = 23) and control (n = 22). During the study, 9 patients in the intervention group and 6 patients in the control group were excluded from the study. At the end of the study, 14 patients in the intervention group and 16 patients in the control group remained in the study analysis. Children assigned to the control group followed the usual care recommended by their primary care physician. Those assigned to the intervention group (both parents and child) participated in an interactive education program about asthma.

### Participants

Children aged between 6 and 12 years with active asthma (symptoms of asthma or treatment for asthma in the previous year) who were registered in our tertiary referral asthma clinic were enrolled. The inclusion criteria were children aged 6–12 years, presence of their parents, diagnosis of mild to severe asthma by the attending physician, no specific physical or mental disease, ability to fill out the questionnaires in Farsi, and willingness of the child and parents to participate in the educational empowerment program. The exclusion criteria were asthma recurrence (acute asthma attacks) and hospitalization.

### Family Empowerment

The family empowerment program consisted of self-directed educational material, lectures (a nurse-focused format), group interaction (a learner-focused format), group discussions, and demonstration of educational films. In this program, participants received information about asthma, its prevalence in children, etiological and trigger factors and how to prevent them, control of asthma triggers both indoors and outdoors, the most common asthma medications, their mechanism of action, the correct use of inhalers and respiratory supplements, instruction of pursed-lip breathing during asthma attacks, how to use a peak flow meter to manage asthma at home, and how to use a practical asthma guide. This program was designed based on the requirements, concerns, and weaknesses of children with asthma and their families regarding knowledge about asthma control and management. The intervention was performed in the clinic by pediatric nurses who were experienced in the education of children with asthma. Nutritional consultation and education was performed in the intervention group by a nutritionist.

### Primary Outcome

The primary outcome measure was change in asthma control. The data collection tools were two questionnaires, one for demographic information and one for asthma control. The questionnaire for asthma control was the Childhood Asthma Control Test (C-ACT) for 4–11-year-old children designed by Liu et al. (13). The validity and reliability (Cronbach’s alpha; α = 0.632) of the questionnaire were tested.

The questionnaires were filled out pre- and post-test, and pre- and post-intervention (family empowerment). The questionnaire consists of 5 questions each scored from 0–4. The mean of the scores is rated as the final score of asthma control. This is the first time that this questionnaire has been validated in Iran.

### Ethics Declaration

The study was reviewed and approved by the University Ethics Committee and performed in accordance with ethical standards. Information about the study was provided comprehensively both orally and in written form to the caregiver adult. Parents and children gave their informed written consent prior to their inclusion in the study.

### Statistical Analysis

Frequency and percentage were used to describe qualitative data, and mean and standard deviation values were calculated for quantitative data. If the data was normally distributed, an independent t-test or paired t-test was applied. For data with abnormal distributions, the Mann-Whitney U test and Wilcoxon Signed-Rank test were used for analysis. P < 0.05 was considered significant and data were analyzed using SPSS version 20.

## RESULTS

In this study, 45 patients were enrolled and randomly divided into two groups: intervention (n = 23) and control (n=22). Demographic variables including age and sex were not significantly different between the two groups in the study (Table 1). Other intervening demographic variables including family history of asthma, maternal university education, homemaker mothers, asthma attacks during the last month, hospital admission during the last month, and taking inhalational therapies were not significantly different between the two groups (Table 1).

**Table 1.** Demographic characteristics of pediatric patients in intervention and control group.

	**Intervention (n = 23)**	**Control (n = 22)**	**P-value**
Age (years)	9.57±1.38	7.86±1.75	0.12^*^
Sex (Male/Female)	16 (69.6%)/7(30.4%)	12 (54.5%)/10(45.5%)	0.29^*^
Mean duration of disease (years)	3.67±2.7	4.63±2.87	0.97^*^
Family history of asthma (%)	38.1%	28.1^¥^	0.51^¥^
Maternal university education (%)	17.4%	18.2%	0.99^¥^
Housewife mothers (%)	73.9%	85.7%	0.48^¥^
Asthma attacks(/last month)	1.08±1.59	1.95±2.45	0.11^*^
Hospital admission (/last month)	0.43±0.95	0.59±0.79	0.23^*^
Inhalational therapies (%)	100%	95.2%	0.47^¥^

* t-test,

¥ Chi-square test

### Primary Outcome

There were no significant differences in asthma control scores at pre-test between the intervention and control groups (p=0.82). However, there was a significant difference in asthma control scores between the intervention and control groups at post-test (p<0.001) (Table 2).

**Table 2.** Asthma control score (C-ACT score) in pre and post-test in intervention and control group.

C-ACT score	Intervention (n = 23)	Control (n = 22)	P-value^*^
Pre-test	2.39±0.55	2.36±0.74	0.82
Post-test	3.61±0.26	3±0.42	<0.001

* t-test

**Figure 1.**
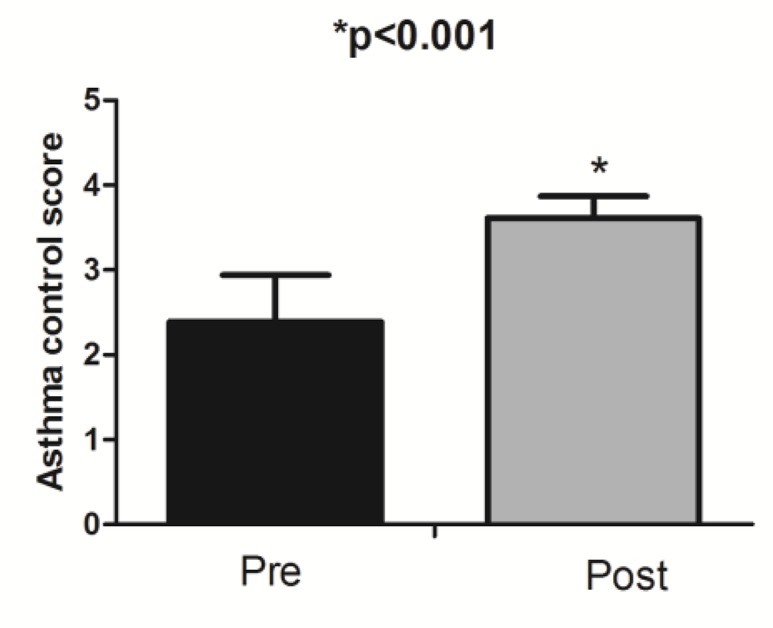
Asthma control score in intervention group in pre and post test.

Interestingly, in the intervention group, where children experienced family empowerment, asthma control scores were significantly higher at post-test compared to pre-test (p<0.001) (Figure 1).

**Figure 2.**
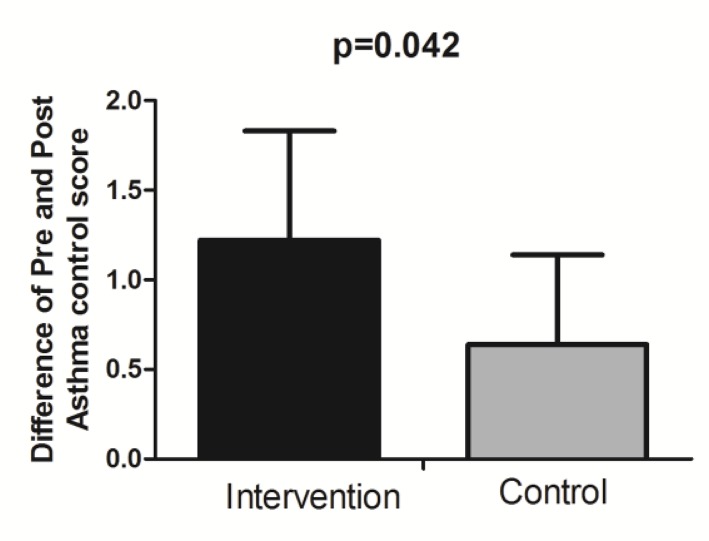
Difference of pre and post-test asthma control score in intervention and control groups.

Differences between asthma control scores at post-test minus pre-test were significantly higher in the intervention group compared to the control group (p=0.042) (Figure 2).

## DISCUSSION

In this study, we focused on family empowerment in school-age children with asthma to determine its efficacy in terms of asthma control. Our results showed that family empowerment in children with asthma aged 6–12 years could have a positive effect on asthma control. Family empowerment is not a part of regular care for asthma patients in many countries including Iran; however, it could be of paramount importance at this age.

In our study, a family empowerment course improved asthma control in school-age children with asthma. Children in the intervention group made significantly fewer visits to the emergency department compared to those in the control group in our study. To assess the improvement, we used the C-ACT questionnaire, which incorporates several items on asthma control. The mean asthma control score was significantly higher in patients who had the intervention (family empowerment) compared to the control group. The asthma control score was not significantly different between the two groups at pre-intervention. This significant improvement in asthma control underlines the importance of family empowerment at school age. The findings of Espinoza-Palma et al. (14) showed that asthma education with or without a self-management plan during asthma hospitalization is effective in reducing future exacerbations, emergency visits, oral steroid use, and hospitalizations.

There is still a challenge whether family empowerment at this age should include both parents and children or children alone. Our family empowerment courses included lectures, group discussions, and demonstration of educational films to both. In asthmatic children and their families, such model facilitates memory retention and increases comfort with future decisions related to their asthma management (15). In establishing this model, Watson et al. started an interactive education program about asthma in children and their parents who visited an emergency department for asthma exacerbation (16). They examined changes in the number of visits to the emergency department during the year after the intervention. In another study, Cano-Garcinuño and colleagues showed that the benefits are apparent when education was aimed at children; however no additional benefit was obtained if the intervention was also aimed at their caregivers (17). This evidence challenges the importance of providing a complete asthma education plan for both patient and family members.

Anticipating the prognosis of asthma is particularly difficult in pediatric groups; however, several factors have been proved to aggravate or assist asthma control at this age. Cigarette smoking is one aggravating factor for asthma control. Besides demographic characteristics, indoor and outdoor triggers could severely change the course of asthma and control level. Many children with asthma continue to experience poorly controlled asthma despite the substantial use of daily controller medication, which reduces their quality of life (18). Therefore, education regarding these triggers and how to prevent them are of paramount importance at this age. In a similar study by Fanelli et al. (19), the authors showed that exercise training has a beneficial role for asthma control in children with asthma.

An asthma registry program could be of great assistance to family empowerment planning. While the demographic aspects of different variants of asthma are not well known, it seems necessary to uncover them by regular follow-ups in order to discover pitfalls in patient’s education program. Recent studies indicate the need for a data registry of demographic variables, labs, and asthma triggers during follow-ups of asthma patients in a respiratory referral center (20).

On the other hand, our study emphasizes the role of nursing in educating the family and children at outpatient clinics of hospitals and at home. This underlines the fact that health services should train asthma-educated nurses as complementary staff to physicians. They can evaluate, plan, and monitor family empowerment more practically. Asthma nurses could combine several teaching strategies for parents and facilitate the completion of individualized asthma education plans (21). The implementation of family empowerment plans would be helpful in involving parents in the therapy of their children with asthma. Training caregivers and changing their quality of life could reflect changes in children with their asthma control (22).

## CONCLUSION

In conclusion, family empowerment significantly improved asthma control in school-age pediatric patients. This program could be proposed for proper asthma control and complication-reducing management of the disease. This program is recommended more broadly for other age groups.
